# Associations of AT(N) biomarkers with neuropsychiatric symptoms in preclinical Alzheimer’s disease and cognitively unimpaired individuals

**DOI:** 10.1186/s40035-021-00236-3

**Published:** 2021-03-31

**Authors:** Kok Pin Ng, Hui Chiew, Pedro Rosa-Neto, Nagaendran Kandiah, Zahinoor Ismail, Serge Gauthier

**Affiliations:** 1 Department of Neurology, National Neuroscience Institute, Singapore, Singapore; 2 Duke-NUS Medical School, Singapore, Singapore; 3 Lee Kong Chian School of Medicine – Imperial College London, Nanyang Technological University, Singapore, Singapore; 4 The McGill University Research Centre for Studies in Aging, Montreal, Canada; 5 Hotchkiss Brain Institute and O’Brien Institute for Public Health; Departments of Psychiatry, Clinical Neurosciences, and Community Health Sciences, University of Calgary, Calgary, Alberta Canada

**Keywords:** Neuropsychiatric symptoms, Preclinical Alzheimer’s disease, Mild behavioral impairment, Amyloid-β, Hyperphosphorylated tau, Neurodegeneration

## Abstract

The development of in vivo biomarkers of Alzheimer’s disease (AD) has advanced the diagnosis of AD from a clinical syndrome to a biological construct. The preclinical stage of AD continuum is defined by the identification of AD biomarkers crossing the pathological threshold in cognitively unimpaired individuals. While neuropsychiatric symptoms (NPS) are non-cognitive symptoms that are increasingly recognized as early manifestations of AD, the associations of NPS with AD pathophysiology in preclinical AD remain unclear. Here, we review the associations between NPS and AD biomarkers amyloid-β (Aβ), tau and neurodegeneration in preclinical AD and cognitively-unimpaired individuals in 19 eligible English-language publications (8 cross-sectional studies, 10 longitudinal, 1 both cross-sectional and longitudinal). The cross-sectional studies have consistently shown that NPS, particularly depressive and anxiety symptoms, are associated with higher Aβ. The longitudinal studies have suggested that greater NPS are associated with higher Aβ and cognitive decline in cognitively unimpaired subjects over time. However, most of the studies have either cross-sectionally or longitudinally shown no association between NPS and tau pathology. For the association of NPS and neurodegeneration, two studies have shown that the cerebrospinal fluid total-tau is linked to longitudinal increase in NPS and that the NPS may predict longitudinal metabolic decline in preclinical AD, respectively. However, evidence for the association between atrophy and NPS in preclinical AD is less consistent. Therefore, future longitudinal studies with well-designed methodologies and NPS measurements are required not only to determine the relationship among AT(N) biomarkers, NPS and cognitive decline, but also to elucidate the contribution of comorbid pathology to preclinical AD.

## Introduction

Alzheimer’s disease (AD) is the most common neurodegenerative disease, which is characterized by core neuropathological features of amyloid plaques and neurofibrillary tangles that result in synaptic loss, neurodegeneration, and cognitive and behavioural manifestations [1]. The diagnosis of AD has traditionally been defined as possible or probable based on clinical syndromes and a definite diagnosis can only be made at autopsy [2, 3]. However, the development of in vivo biomarkers to identify amyloid-β (Aβ) (A) deposition, pathologic tau (T), and neurodegeneration (N) has made it possible to measure AD pathophysiology in living persons. In this regard, AT(N) is an unbiased descriptive classification scheme that groups Aβ plaques, fibrillar tau and neurodegeneration biomarkers that are available in AD research and clinical applications [4]. Hence, the definition of AD has been shifted from a clinical syndrome to a biological construct as proposed in the 2018 National Institute on Aging—Alzheimer’s Association (NIA-AA) Research Framework, where a combination of syndromal cognitive staging (cognitively unimpaired, mild cognitive impairment [MCI] and dementia) and biomarker profiles (A+T−(N)−: Alzheimer’s pathologic change; A+T+(N) − and A+T+(N)+: Alzheimer’s disease; A+T−(N)+: Alzheimer’s and concomitant suspected non-Alzheimer’s pathologic change) forms the biological Alzheimer’s continuum [5].

The identification of AD biomarkers in the AT(N) classification scheme that cross the pathological threshold in cognitively unimpaired individuals has led to the conceptual framework of a preclinical stage in the AD continuum [6, 7]. This concept is further validated in presymptomatic autosomal dominant AD mutation-carriers where in vivo pathophysiological markers are elevated years prior to the onset of symptoms [8]. Given the hypothesis that early intervention may offer the greatest chance of treatment success, preclinical AD is currently a main focus in AD clinical trials [9–11]. It has been postulated that the pathophysiology of AD has a pattern of temporal evolution, starting with Aβ plaques and fibrillar tau, followed by neuronal dysfunction as the eventual pathway, leading to cognitive impairment [12, 13]. This hypothesis has been supported by a study in preclinical AD which showed that Aβ and hyperphosphorylated tau aggregates synergistically interact to cause a downstream metabolic decline in brain networks affected early in AD [14]. Although amnestic presentation is the most prominent and widely reported cognitive deficit in AD, non-amnestic presentations such as behavioral change are increasingly recognized as early manifestations of AD [15–18]. Given that neuropsychiatric symptoms (NPS) may be the first manifestation of AD rather than cognition in nature, further studies are needed to evaluate where NPS fit in the AT(N) pathophysiological pathway.

### NPS as manifestations of preclinical AD

NPS, including behavioral and psychiatric symptoms, are frequently reported by AD patients [19–21] and are associated with poorer outcomes in cognition, functional status and quality of life, and faster disease progression to severe dementia [22–25]. NPS, particularly anxiety, apathy and depressive symptoms [26–30], are also frequently observed at preclinical and prodromal/MCI stages of AD and may predict progression to dementia compared to those without NPS [23, 26, 31]. Therefore, to systematically study early NPS in neurodegenerative diseases, mild behavioral impairment (MBI) has been proposed as a neurobehavioral construct characterized by later-life emergent and sustained NPS as an at-risk state for incident cognitive decline and dementia. Examination of the associations of AD pathophysiology with NPS and MBI in preclinical stages of the AD continuum [32] may provide insights into the neurobiological basis of NPS as an early non-cognitive manifestation of AD [33, 34].

### MBI

The new MBI criteria proposed by the ISTAART (International Society to Advance Alzheimer's Research and Treatment) Neuropsychiatric Symptoms Professional Interest Area in 2015 have been increasingly validated as a neurobehavioral syndrome that could be used to identify individuals at a risk of developing dementia, who may or may not have cognitive symptoms. The core criteria include emergent and persistent behavioral or personality changes starting after 50 years of age and persisting for ≥6 months that are of sufficient severity leading to impairment in interpersonal relationships, social functioning or the ability to perform in workplace [35]. Importantly, MBI represents a neurobehavioral risk axis that serves as a complement to the traditional neurocognitive risk axis represented by subjective cognitive decline (SCD) and MCI. Importantly, the two axes are not mutually exclusive – MBI can manifest before cognitive symptoms appear (i.e., at the normal cognition stage), or in conjunction with cognitive symptoms (at the SCD or MCI stage), and the risk of cognitive decline is greater in the presence of symptoms from both cognitive and behavioral axes, compared to either axis alone [36]. The MBI diagnostic criteria standardize the assessment of NPS in pre-dementia populations and support the early identification of neurodegenerative illness through behavioral manifestations, so that symptomatic treatment or future disease-modifying agents may be used promptly to improve clinical outcomes.

The Mild Behavioral Impairment Checklist (MBI-C) [37] has been specifically developed as a MBI case ascertainment instrument, which also allows for the monitoring of MBI symptoms over time. In a primary care validation study, the prevalence of MBI was 14.2% in MCI using a cut-off point of 6.5, and 5.8% in SCD using a cut-off point of 8.5 [38, 39]. In a cohort of 9931 older adults who did not have MCI or dementia at baseline, a comprehensive neuropsychological battery was measured at baseline and 1 year later while the MBI-C was administered 1 year later. Ten percent of the participants had MBI and they showed significantly worse cognitive performance at baseline and significantly greater decline over 1 year in attentional intensity, sustained attention, attentional fluctuation and working memory [40]. These data also suggest that MBI, identified at the stage of SCD or before, may be an early marker of neurodegenerative diseases.

## Methods

To review the associations between NPS and AD pathophysiology (Aβ, hyperphosphorylated tau and neurodegeneration) in preclinical AD and cognitively unimpaired individuals, we conducted a PubMed search by August 31, 2020, using combinations of the following keywords: “Alzheimer”, “psychiatric”, “neuropsychiatric”, “behavior”, “mood”, “affective”, “psychosis”, “depression”, “apathy”, “prodromal”, “preclinical”, “asymptomatic”, “amyloid”, “tau”, “neurodegeneration”, “atrophy”, “PET”, “FDG”, and “fluorodeoxyglucose”. References of the included publications were also screened. Articles were included if they met the following 5 criteria:

1. The study population consisted of participants who:

(i) met the criteria for preclinical AD as defined by the NIA-AA Research Framework [5], or
(ii) were cognitively unimpaired without preclinical AD – defined participants who scored within normal limits on baseline cognitive testing or neuropsychological assessment or had a Clinical Dementia Rating (CDR) score of 0. However, studies were excluded if the study population primarily involved participants with psychiatric diagnoses or if the study specifically recruited participants with psychiatric disorders when evaluating cognition and/or biomarkers.

2) The study assessed the presence or severity of NPS. NPS were measured by the 12 items of Neuropsychiatric Inventory (NPI), including delusions, hallucinations, anxiety, depression, agitation/aggression, euphoria, disinhibition, irritability/lability, apathy, aberrant motor activity, night-time behavioural disturbances, and appetite/eating abnormalities [41]. Studies were also included if validated self- or informant-reported rating scales were used for assessment of NPS, such as the Neuropsychiatric Inventory Questionnaire (NPI-Q), MBI-C and Geriatric Depression Scale (GDS) [42].
3) The study evaluated one or more of the AT(N) biomarkers:

(i) A: amyloid deposition using amyloid positron emission tomography (PET), cerebrospinal fluid (CSF) amyloid or plasma amyloid levels,
(ii) T: tau deposition using tau-PET or CSF phosphorylated tau levels, and
(iii) N: glucose metabolism, atrophy or CSF  total-tau levels.

4) The study examined the association between NPS and AT(N) biomarkers.
5) The study was published in English.

Two authors (KPN and HJC) independently conducted the search, and reviewed articles for selection. Data extraction was performed using a standardized form. Study quality was assessed using the Newcastle-Ottawa Scale [43, 44]. Any disagreement was resolved by consensus between the two authors.

## Results

A total of 3042 English-language abstracts were screened and 41 full-text articles were assessed for inclusion. Of these, 14 were excluded for including subjects with MCI and/or dementia, 5 were excluded for not reporting the outcome of interest and 3 were excluded for primarily including subjects with depression. Finally, 19 articles were included in the review (Tables 1 and 2).

**Table 1:** Characteristics of the reviewed cross-sectional studies

No.	Author	Study population (cohort)	AT(N) Biomarkers	Biomarker method / assay	NPS instrument	NPS with association	Significant findings
1	Binette et al. 2020 [45]	117 ADAD mutation carriers (DIAN)	A	PiB-PET, global SUVR	IPIP-NEO-120, NPI-Q	NPI-Q score	• ↑ A associated with ↑ NPI-Q score
2	Gatchel et al. 2017 [46]	111 CU - 5 with depression (HABS)	A T	PiB PET, DVR cut-off > 1.20 [^18^F]T807-PET	GDS	GDS score	• ↑ T associated with ↑ GDS score
3	Kim et al. 2018 [47]	667 CU (NACC)	A T	Pathology: neuritic plaques (T) and neurofibrillary tangles (A)	NPI-Q	Psychosis	• ↑ T associated with ↑ psychosis • No association between A and NPI-Q score
4	Krell-Roesch et al. 2017 [48]	1038 CU (MCSA)	A	PiB-PET, SUVR cut-off ≥1.40	BAI, BDI-II	Anxiety symptoms, depressive symptoms	• ↑ A associated with ↑ anxiety and ↑ depressive symptoms
5	Lussier et al. 2020 [49]	96 CU (TRIAD – McGill University)	A T N (GM volume)	[^18^F]AZD4694-PET, SUVR cut-off by visual inspection [^18^F]MK6240-PET 3 T MRI	MBI-C	MBI-C score	• A linearly correlated with total MBI-C score • No association between T, N and MBI-C score
6	Sun et al. 2008 [50]	995 CU and MCI - 348 depressed (NAME cohort)	A	Sandwich Aβ ELISA	CES-D	Depression	• ↑ A associated with depression *(findings likely driven by subjects with MCI)*
7	Yasuno et al. 2016 [51]	30 CU	A	PiB-PET, BP_ND_ categorized by tertiles	GDS-S	GDS-S score	• ↑ A associated with ↑ GDS-S score
8	Krell-Roesch et al. 2016 [52]	668 CU (MCSA)	N (Glucose metabolism)	FDG-PET, SUVR cut-off < 1.32 in AD-signature ROI	NPI-Q, BAI, BDI-II	Depression, anxiety	• ↓ Glucose metabolism associated with depression, especially in APOE ε4 carriers • ↓ Glucose metabolism associated with ↑ depression and ↑ anxiety symptoms
9	Donovan et al. 2015 [53]	248 CU (HABS)	N (HV, glucose metabolism) A	3 T MRI, FDG-PET PiB-PET, DVR cut-off > 1.20	GDS	Depression	• ↓ HV and ↓ glucose metabolism associated with ↑ GDS score • ↓ HV associated with ↑ dysphoria and ↑ apathy-anhedonia scores • ↓ Glucose metabolism associated with ↑ apathy-anhedonia scores • No association between A and GDS score

↑: higher/greater; ↓ lower/lesser; A: amyloid-β; Aβ: amyloid-β; Aβ_40_: amyloid-β 1–40; Aβ_42_: amyloid-β 1–42; *ADAD* autosomal dominant Alzheimer’s disease, *BAI* Beck Anxiety Inventory, *BDI-II* Beck Depression Inventory-II, *BP*_*ND*_ binding potential (BP) estimates relative to nondisplaceable (ND) binding, *CES-D* Center for Epidemiological Studies Depression Scale, *CU* cognitively unimpaired, *DIAN* Dominantly Inherited Alzheimer Network, *DVR* distribution volume ratio, *ELISA* enzyme linked immunosorbent assay, *FDG* fluorodeoxyglucose, *GDS* geriatric depression scale, *GDS-S* geriatric depression scale short form, *GM* grey matter, *HABS* Harvard Aging Brain Study, *HV* hippocampal volume, *IPIP-NEO-120* International Personality Item Pool Representation of the NEO PI, *MBI-C* Mild Behavioural Impairment Checklist, *MCSA* Mayo Clinic Study of Aging, *MRI* magnetic resonance imaging, *N* neurodegeneration, *NACC* National Alzheimer’s Coordinating Center, *NAME* Nutrition, Aging and Memory in the Elderly study, *NPI-Q* Neuropsychiatric Inventory Questionnaire, *OR* odds ratio, *PET* positron emission tomography, *PiB* Pittsburgh Compound B, *ROI* region of interest, *T* tau, *SUVR* standardized uptake value ratio, *TRIAD* Translational Biomarkers of Aging and Dementia cohort

**Table 2:** Characteristics of the reviewed longitudinal studies

No.	Author	Study population (cohort)	Follow-up duration	AT(N) Biomarkers	Biomarker method / assay	NPS instrument	NPS with association	Significant findings
1	Binette et al. 2020 [45]	115 CU at-risk for AD (PREVENT-AD)	3 years	A T	[^18^F]NAV4694-PET, global SUVR [^18^F]AV1451-PET, SUVR in Braak stages I, III, IV	GDS-S, AES, Geriatric anxiety scale, Stress subscale, Big5 inventory	Apathy, anxiety	• ↓ A associated with ↓ anxiety and apathy at baseline • ↓ T associated with ↓ apathy at baseline • No longitudinal association between A, T and NPS
2	Babulal et al. 2016 [54]	118 CU at baseline (9 with mood disorder) 66 CU on follow-up (Washington University)	1 year	A T N (CSF total tau)	PiB-PET, MCBP cut-off used median split CSF Aβ_42_, total tau, ptau181 ELISA. Cut-off values used median split	POMS-SF, GDS-S, NPI-Q	Total mood disturbance, NPI-Q score, anxiety and depressive symptoms	• ↑ A longitudinally associated with ↑ total mood disturbance, anxiety, depression scores on POMS-SF, ↑ NPI-Q score and ↑ GDS-S score • T and N predicted longitudinal changes in NPI-Q score
3	Blasko et al. 2010 [55]	331 CU (VITA cohort)	2.5 years	A	Sandwich Aβ ELISA	DSM-IV criteria, GDS	Depression	• ↑ A at baseline predicted late-onset depression
4	Direk et al. 2013 [56]	657 nondemented (Rotterdam study)	11 years	A	Sandwich Aβ ELISA	CES-D, HADS	Depressive symptoms	• ↑ A associated with ↑ depressive symptoms at baseline and subsequent incident dementia
5	Donovan et al. 2018 [57]	270 CU (HABS)	5 years	A	PiB-PET, DVR as continuous variable	GDS	Anxious-depressive symptoms	• ↑ A longitudinally associated with ↑ GDS and anxiety-concentration scores
6	Gatchel et al. 2019 [58]	276 CU - 22 with mild depression (HABS)	1 year	A N (HV, glucose metabolism)	PiB-PET, DVR cut-off > 1.20 3 T MRI, FDG-PET	GDS	GDS score	• ↑ A moderated the longitudinal association between ↑ depressive symptoms and ↑ cognitive decline
7	Harrington et al. 2017 [59]	359 CU (AIBL)	54 months	A	PiB-PET, SUVR cut-off ≥1.5	GDS-S	Incident depression	• ↑ A longitudinally associated with ↑ incident depression
8	Johansson et al. 2020 [60]	104 CU 53 MCI (BioFINDER)	4 years	A N (GM thickness, subcortical volumes)	[^18^F] flutemetamol PET, SUVR cut-off > 1.42 3 T MRI	AES, HADS	Apathy, anxiety	• ↑ A longitudinally associated with ↑ apathy, ↑ anxiety over time • ↑ N longitudinally associated with ↑ apathy over time (in CU and MCI subjects)
9	Perin et al. 2018 [61]	585 CU (AIBL)	72 months	A	PiB-PET, SUVR cut-off ≥1.5	GDS-S	GDS-S score, apathy-anxiety symptoms	• ↑ A longitudinally associated with ↑ GDS-S score and apathy-anxiety symptoms
10	Qiu et al. 2015 [62]	223 CU - 68 depressed (NAME cohort)	6.2 years	A	Sandwich Aβ ELISA, Aβ + cut-off used median split of plasma Aβ_40_/Aβ_42_ ratio	CES-D	Nil	• No association between A and depression
11	Ng et al. 2017 [63]	33 preclinical AD – A + T+ 60 at-risk for AD - A + T- or A-T+ 22 healthy control (ADNI)	2 years	N (Glucose metabolism)	FDG-PET	NPI	NPI-Q score	• ↑ NPI-Q score predicted longitudinal ↓ glucose metabolism in preclinical AD

↑: higher/greater; ↓ lower/lesser, *A* amyloid-β; *AD* Alzheimer’s disease, *ADNI* Alzheimer’s Disease Neuroimaging Initiative, *AES* Apathy Evaluation Scale, *AI* anterior insula, *AIBL* Australian Imaging, Biomarker & Lifestyle Study of Ageing, *Aβ* amyloid-β, *Aβ*_*40*_ amyloid-β 1–40; *Aβ*_*42*_ amyloid-β 1–42, *CES-D* Center for Epidemiological Studies Depression Scale, *CSF* cerebrospinal fluid, *CU* cognitively unimpaired, *DSM-IV* Diagnostic and Statistical Manual of Mental Disorders-IV, *DVR* distribution volume ratio, *ELISA* enzyme linked immunosorbent assay, *FDG* fluorodeoxyglucose, *GDS* geriatric depression scale, *GDS-S* geriatric depression scale short form, *GM* grey matter, *HABS* Harvard Aging Brain Study, *HADS* Hospital Anxiety and Depression Scale, *HV* hippocampal volume, *MCBP* mean cortical binding potential, *MCI* mild cognitive impairment, *N* neurodegeneration, *NAME*, Nutrition, Aging and Memory in the Elderly study, *NPI* Neuropsychiatric Inventory, *NPI-Q* Neuropsychiatric Inventory Questionnaire, *OR* odds ratio, *PCC* posterior cingulate cortex, *PET* positron emission tomography *PiB* Pittsburgh Compound B, *POMS-SF* Profile of Mood States - Short Form, *ptau181* phosphorylated tau181, *SUVR* standardized uptake value ratio, *T* tau, *VITA* Vienna Transdanube Aging study, *vmPFC* ventromedial prefrontal cortex

The majority (17/19, 89.5%) of studies involved cognitively unimpaired participants who did not meet the biological definition for preclinical AD. Only one study recruited preclinical AD subjects as defined by the NIA-AA research framework [63]. In addition, one study included 2 different cohorts of cognitively unimpaired subjects – autosomal-dominant AD mutation-carriers from the Dominantly Inherited Alzheimer Network (DIAN) and subjects at a high risk of progression to AD from the PREVENT-AD (PRe-symptomatic EValuation of Experimental or Novel Treatments for AD) cohort [45].

Eight (42.1%) of the studies were cross-sectional, 10 (52.6%) were longitudinal and 1 (5.3%) employed two different cohorts consisting of both cross-sectional and longitudinal data. Furthermore, 8 (42.1%) studies evaluated multiple NPS, using either a single tool such as the NPI-Q or MBI-C, or a combination of tools. Eleven (57.9%) studies evaluated only depression or depressive symptoms while 1 (5.3%) evaluated psychosis. Seventeen studies (89.5%) evaluated the associations of Aβ with NPS (7 cross-sectional, 9 longitudinal, and 1 mixed), 5 (26.3%) evaluated the associations of tau with NPS (3 cross-sectional, and 2 longitudinal) and 7 (36.8%) evaluated the associations of neurodegeneration (brain atrophy, CSF total-tau and glucose metabolism) with NPS (3 cross-sectional and 4 longitudinal). In addition, 8 of them (42.1%) evaluated multiple AT(N) biomarkers (2 A, T and N; 3 A and T; 3 A and N).

### Associations of Aβ with NPS

#### Aβ measured using PET imaging and in CSF

Twelve studies used PET imaging only and one used a combination of PET and CSF modalities. They included 5 cross-sectional and 6 longitudinal studies, and 1 with both cross-sectional and longitudinal data from two different cohorts.

In a cross-sectional study of 117 asymptomatic participants from the DIAN cohort [45], all participants were autosomal AD mutation-carriers (85 *PSEN1*, 17 *PSEN2* and 15 *APP* mutation-carriers) with the mean estimated years to onset age of 12.9 years. There was no significant difference in behavioural features among the three mutation types. A higher baseline NPI-Q score, lower scores on the intellect personality trait and fewer years of education were associated with a higher global Aβ deposition (*r* = 0.37, *P* < 0.001). However, a limitation of this study was that the participants were not controlled for estimated years to onset age of AD. A key strength of this study was the inclusion of participants with autosomal dominant AD mutations. The near-complete penetrance of these pathogenic mutations in the DIAN cohort suggested that the cohort in this study was representative of subjects with preclinical AD [64].

A recent cross-sectional study of 96 cognitively unimpaired elderly subjects has demonstrated a moderate linear correlation of MBI-C score with global Aβ deposition (*r* = 0.27, *P* < 0.0074) and striatal Aβ deposition on amyloid PET (*r* = 0.3, *P* < 0.0028). This study demonstrated for the first time a link between the MBI construct measured using MBI-C and amyloid pathology in preclinical AD [49].

While the above studies evaluated the associations of Aβ with NPS measured by NPI-Q or MBI-C, there are also studies evaluating the associations of Aβ with anxiety or depressive symptoms measured using scales specific for NPS. In this regard, a large cohort study of 1038 subjects from the Mayo Clinic Study of Aging (MCSA) has demonstrated that Aβ deposition is weakly correlated with anxiety symptoms measured on the Beck Anxiety Inventory (odds ratio [OR] = 1.04; 95%CI, 1.01–1.08, *P* = 0.022) and marginally significantly correlated with depressive symptoms measured on the Beck Depression Inventory II (OR = 1.03; 95%CI, 1.00–1.06, *P* = 0.077) [48]. Although the associations were weak, the study may have been underpowered due to a low prevalence of anxiety and depressive symptoms in this population-based cohort – only 6.3% and 7.1%, respectively. One study involving 30 cognitively unimpaired elderly subjects showed an association between Aβ deposition and depressive symptoms measured using the Geriatric Depression Scale Short Form (GDS-S) (*r* = 0.59, *P* = 0.004) [51]. However, the sample size was small and the study was assessed to be of low quality.

Evidence for the association of Aβ with NPS was not consistent across studies, as our review identified 2 cross-sectional studies that did not show a significant association between Aβ deposition and NPS. Two overlapping studies in the Harvard Aging Brain Study (HABS) cohorts failed to show a relationship between depressive symptoms measured on the GDS and Aβ deposition on amyloid-PET at baseline [46, 53], although subsequent longitudinal findings from the HABS revealed a significant association between NPS and Aβ, which will be elaborated below.

Of the 7 longitudinal studies reviewed, 2 have revealed a significant relationship between baseline Aβ positivity, NPS and cognitive decline over time. The first study was conducted in the HABS cohort and showed that over a 1-year period, an increase in depressive symptoms on the GDS was associated with cognitive decline only in the presence of Aβ deposition (β = − 0.19; 95%CI, − 0.27 to − 0.12, *P* < 0.001). Significantly, this association was demonstrated at a lower distribution volume ratio (DVR) threshold of 1.06 than the previously published HABS cut-off of 1.20 [58]. The second study in the Swedish BioFINDER cohort revealed that Aβ deposition correlated with the informant-rated apathy on the Apathy Evaluation Scale (AES) (β = 0.18, *P* = 0.022) and self-rated anxiety on the Hospital Anxiety and Depression Scale (HADS) (β = 0.18, *P* = 0.024), but not with the depressive symptoms [60]. In addition, the anxiety score interacted with the Aβ status to predict cognitive decline on the Mini-Mental State Examination (MMSE) over 4 years. Although this study comprised 104 cognitively unimpaired and 53 MCI participants who were analyzed together, sensitivity analyses controlling for the clinical diagnosis (cognitively unimpaired *versus* MCI) provided similar findings, and further *post-hoc* analysis showed that the informant-rated apathy was only significantly associated with Aβ deposition in cognitively unimpaired participants.

The other four longitudinal studies reviewed have shown an association between baseline Aβ and longitudinal changes in NPS. A recent publication from the HABS cohort has shown that the baseline Aβ deposition is associated with an increase in anxious-depressive symptoms on the GDS over a 5-year period [57]. In one of the two publications from the Australian Imaging, Biomarker and Lifestyle Study of Ageing (AIBL), Perin et al. demonstrated that the baseline Aβ deposition was significantly associated with longitudinal increase in total GDS-S score (*d* = − 0.25; 95%CI, − 0.45 to − 0.05) and apathy-anxiety symptoms (*d* = − 0.28; 95%CI, − 0.48 to − 0.08) over 72 months [61]. The other earlier AIBL study has demonstrated greater incident depression in Aβ-positive participants, but a subsequent reanalysis found that the role of Aβ was overestimated [59, 61]. In another study by Babulal et al. from Washington University that combined amyloid-PET and CSF amyloid biomarkers, a high CSF tau/Aβ_42_ ratio was associated with increased mood disturbance, anxiety and depression on the Profile of Mood States–Short Form (*P* = 0.005, 0.02 and 0.04 respectively), as well as with the NPI-Q total score (*P* = 0.003) at 1-year follow-up. In addition, there were also significant associations between high Aβ deposition and increased GDS (*P* = 0.01) and NPI-Q scores (*P* = 0.04) [54]. However, a limitation of this study was that a median split was used to dichotomize PET and CSF biomarker positivity, thus the patient groups with “high” biomarkers may not represent the true preclinical AD. Binette et al. have shown contrasting results using data from the PREVENT-AD cohort [45]. This study involved 115 participants who did not have preclinical AD but were considered at a high risk of AD as they had a parental or multiple-sibling family history of sporadic AD. While Aβ deposition was demonstrated to be associated with anxiety on the Geriatric Anxiety Scale, apathy on the AES and the personality trait neuroticism at baseline (*r* = 0.23, *P* = 0.013), it did not correlate with changes in NPS over a 3-year follow-up period.

#### Aβ in plasma

Four studies have examined the relationship between plasma amyloid levels (measured using the sandwich enzyme linked immunosorbent assay for Aβ) and depression or depressive symptoms assessed with GDS, HADS or the Center for Epidemiologic Studies Depression Scale (CES-D). The results were inconsistent. A cross-sectional and a longitudinal study showed no correlation [50, 62], while the other longitudinal study showed that higher plasma Aβ_42_ levels at baseline could predict incident depression over 5 years (Wald *χ*^2^ = 6.30, df = 1, *P* = 0.012, OR = 1.8; 95%CI, 1.1–2.8) [55]. One publication from the Rotterdam Study cohort involving 980 participants, of whom 657 were followed-up for up to 11 years, showed that high baseline plasma Aβ_40_ levels were associated with depressive symptoms (OR = 2.22; 95%CI, 1.33–3.73, *P* = 0.002) [56]. Significantly, this association was supported by subjects subsequently developing incident dementia, suggesting that the depressive symptoms may be manifestations of prodromal AD.

#### Aβ measured in pathological studies

We found one pathological study. In this retrospective cross-sectional study of 667 cognitively asymptomatic subjects using data from the National Alzheimer’s Coordinating Center (NACC), higher neuritic plaque load was associated with psychosis measured on the NPI-Q (OR = 2.47; 95%CI, 1.54–3.96) [47]. However, a MMSE score of ≥24 was used to define “cognitively asymptomatic” in the study cohort. Therefore, while findings from this study support the association of Aβ with NPS, it is possible that some of the subjects may have MCI or mild dementia.

### Associations of tau with NPS

#### Tau measured using PET imaging

Two cross-sectional studies have evaluated the associations between tau-PET and NPS, but they provided contrasting results. One from the HABS cohort showed that greater depressive symptoms as reflected by higher GDS scores were associated with greater inferior temporal (partial *r* = 0.188, *P* = 0.050) and entorhinal cortex tau depositions (partial *r* = 0.183, *P* = 0.055), in the absence of an association with Aβ [46]. However, this study may have been underpowered due to the modest sample size (*n* = 111) and the mild severity of depressive symptoms (mean GDS score of 3.93) in the sample. On the other hand, the other study by Lussier et al. did not reveal a significant correlation between MBI measured with MBI-C and tau deposition, despite a positive association with Aβ deposition, although not all participants were Aβ-positive [49].

One report has analyzed longitudinal data from 115 at-risk subjects from the PREVENT-AD cohort. While apathy on the AES and a number of personality traits including higher neuroticism and lower openness and extraversion were associated with tau deposition at baseline (*r* = 0.29, *P* = 0.02) [45], tau was not shown to influence the change in NPS over time. Some have argued that personality changes, traditionally framed in a neurodevelopmental context, may be better framed as neuropsychiatric or behavioral changes consistent with MBI when considered in a neurodegenerative disease context for dementia prognostication. For example, neuroticism may represent emerging affective dysregulation, lower extraversion could represent apathy or decreased initiative and/or interest, and lower openness may represent rigidity as part of MBI impulse dyscontrol [65]. More research is required to explore this interesting issue to understand the association between behavioral and personality changes and AD biomarkers.

#### Tau measured as CSF phosphorylated tau

In the longitudinal study of 118 cognitively unimpaired elderly, Babulal et al. reported no association between CSF p-tau181 levels and NPS at baseline. However, longitudinal data showed a significant correlation between higher CSF p-tau181 levels and increase in NPI-Q score at 1-year follow-up (*P* = 0.05) [54].

#### Tau measured in pathological studies

In the only pathological study using data from the NACC, the higher stage of neurofibrillary tangle pathology was not associated with psychosis in cognitively asymptomatic subjects [47].

### Associations of neurodegeneration with NPS

#### Neurodegeneration measured using CSF total-tau

One longitudinal study has provided data for CSF total-tau. In the study that combined amyloid PET and CSF AT(N) biomarkers, Babulal et al. demonstrated that the CSF total-tau levels could predict changes in NPS, with a significant correlation found between higher baseline CSF total-tau levels and longitudinal increase in NPI-Q score (*P* = 0.001) [54].

#### Neurodegeneration measured using magnetic resonance imaging

Four cross-sectional studies have examined the relationship between brain atrophy and NPS, with mixed findings. A study from the HABS involving 268 non-depressed cognitively unimpaired subjects found that the higher GDS score was associated with lower hippocampal volumes (*P* = 0.03), with secondary models using principal component analysis showing that the association was significant for two subdomains of the GDS (dysphoria and apathy-anhedonia) [53]. In a mixed cohort of 104 cognitively unimpaired and 53 MCI subjects from the BioFINDER cohort, the informant-reported apathy on the AES was associated with atrophy in AD-signature cortical regions, which included the entorhinal, inferior temporal, middle temporal, and fusiform cortices (*P* < 0.05). Both the informant- and the self-reported apathy correlated with smaller hippocampal and nucleus accumbens volumes (β = − 0.242 to − 0.273, *P* = 0.02–0.025) [60]. However, the absence of subgroup data for cognitively unimpaired subjects has limited the interpretation of these results in the context of this review.

Two other studies have reported a null association between brain atrophy and NPS. In the study evaluating MBI and AT(N) biomarkers in cognitively unimpaired subjects, no significant association was found between grey matter volumes and MBI-C scores [49]. In another study, Gatchel et al. found that the hippocampal volumes did not moderate the association between worsening depressive symptoms and cognitive decline, while Aβ deposition did [58].

#### Neurodegeneration measured using fluorodeoxyglucose (FDG)-PET

Four studies have examined the relationship between glucose metabolism and NPS, including 3 cross-sectional and 1 longitudinal.

One of the cross-sectional studies from the MCSA has demonstrated an association between glucose hypometabolism on FDG-PET and depression measured on the NPI-Q (OR = 2.12; 95%CI, 1.23–3.64; *P* ≤ 0.05), especially in apolipoprotein E4 (ApoE4) carriers (OR = 2.59; 95%CI, 1.00–6.69; *P* = 0.05) [52]. Another cross-sectional study from the HABS has shown a marginally significant association between higher GDS score – driven by the anxiety-concentration subdomain – and lower glucose metabolism (*P* = 0.06) [53].

The longitudinal study of subjects from the Alzheimer’s Disease Neuroimaging Initiative (ADNI) cohort [63] involved 115 cognitively normal subjects who were stratified into three groups: preclinical AD (with presence of both Aβ and tau pathologies), asymptomatic at risk of AD (amyloid or tau pathology present) and healthy controls. In the cohort of 33 preclinical AD subjects, higher NPI-Q scores were associated with higher glucose metabolism in the posterior cingulate cortex, ventromedial prefrontal cortex and anterior insula at baseline. Significantly, the higher NPI-Q scores, predominantly in the sleep/night time behaviour disorders and irritability/liability components, could predict the subsequent decline in global glucose metabolism over 2 years (β = 0.52, *P* = 0.01) [63]. No longitudinal correlation with cognitive decline was found. This was the only study in a preclinical AD cohort as defined by the AT(N) framework to show evidence for an association between NPS and subsequent neurodegeneration.

## Discussion

In this review, we have found fairly consistent evidence for the cross-sectional correlation of NPS - in particular depressive symptoms - with Aβ. Emerging evidence has also suggested that Aβ is associated with longitudinal increase in NPS and might moderate the relationship between NPS and cognitive decline [54, 57–61]. However, the strength of the correlations identified was weak, even in studies in large cohorts, in which the statistical power was likely limited by the low prevalence of NPS. Recent findings from the HABS and BioFINDER cohorts showing that Aβ moderates the relationship between NPS (depression or anxiety) and cognitive decline are promising [58, 60]. Notably, in the HABS cohort, this effect occurred at a lower Aβ DVR threshold than previously published cut-offs [58]. This suggests that Aβ and NPS may have a synergistic relationship in the very early stages of AD pathology, and some NPS may be an early manifestation of AD pathology.

Compared to Aβ, evidence supporting a link between tau pathology and NPS in preclinical AD is currently lacking. Cross-sectional studies have provided conflicting evidence for the associations of tau with NPS, although in one study, higher CSF p-tau181 levels were shown to be associated with an increase in NPI-Q score at 1-year follow-up. This may reflect the prevailing hypothesis that some NPS are manifestations of early AD pathology, before the presence of significant tau deposition. This is supported by findings of Gatchel et al. from the HABS cohort of an association between depressive symptoms and tau deposition in the entorhinal cortex, which is one of the early regions demonstrating tangle pathology in AD [46, 66]. More studies are needed to clarify the associations of tau with MBI measured with the MBI-C, using tau PET and possibly plasma tau isoforms in the future [67, 68].

The evidence for the associations between neurodegeneration and NPS in preclinical AD is inconsistent, which may be due to the small number of studies and the variability of biomarkers studied. However, studies have extensively evaluated the neuroanatomical and metabolic correlates of NPS in the MCI and dementia stages of AD, suggesting that the NPS are associated with neurodegeneration of specific neural networks in AD [69–71]. Therefore, it may not be surprising to observe a lack of an association between NPS and neurodegeneration in preclinical AD. On the other hand, recent evidence has suggested that NPS, such as sleep/night time behaviour disorders and irritability/liability, may predict subsequent hypometabolism in subjects with definite preclinical AD [63]. In another study of non-demented participants, new-onset MBI is associated with greater increases in plasma neurofilament light, a validated biomarker of axonal damage seen in neurodegeneration, over 2 years [72]. Based on the hypothetical model of the pathological cascade in AD [73], where Aβ biomarkers become abnormal first, followed by neurodegenerative biomarkers and cognitive symptoms, the findings of an association between NPS and amyloid in preclinical AD may support the emerging conceptual framework that specific NPS constitute an early clinical manifestation of AD pathophysiology. However, longitudinal studies with various biomarkers of neurodegeneration are needed to validate this hypothesis.

It is, however, premature to assume the causality among AT(N) biomarkers, NPS and cognitive decline, and the specificity of the NPS, given the overlap between depression, anxiety and apathy symptoms in the studies. In the 11 longitudinal studies we reviewed, the presence of AT(N) biomarkers was cross-sectionally determined at baseline, with no correlation with repeated measures performed. Meanwhile, the pathological studies have shown that the entity of “pure” AD is uncommon, and coexistent proteinopathies are found in 65% of pathologically confirmed AD. Therefore, while Aβ and/or tau accumulation may herald the start of AD pathology, the neuropathological mechanisms that lead to NPS and subsequent cognitive decline may not be solely amyloid- or tau-dependent. The contribution of comorbid pathologies such as Lewy body or vascular disease cannot be excluded. Indeed, a study in subjects with late-life depression has shown that atrophy in the BA36 region of the perirhinal cortex is associated with a greater burden of vascular risk factors (measured by the Framingham Stroke Risk Scale) and the volume of cerebral white matter hyperintensity [74]. Future studies should therefore not only measure the longitudinal changes in AT(N) biomarkers in relation to NPS and cognitive decline, but also the role of comorbid pathology.

Another intriguing question is whether treatment of NPS in preclinical AD may reduce the risk of progression to dementia. None of the studies we reviewed were designed to adequately address this. In the study by Gatchel et al., 14.9% of the subjects were treated with antidepressants; however, this did not modify the relationship between depression and cognitive decline [58]. While mouse studies have suggested that the treatment with antidepressants may modulate Aβ levels or even reduce Aβ plaques, evidence in human studies is just starting to arise. In this regard, a recent prospective study has shown an overall 9.4% reduction in CSF Aβ_42_ levels in cognitively normal older adults treated with short-term longitudinal doses of escitalopram compared to those receiving placebo [75]. In addition, a study using ADNI data found that MCI subjects with a remote history of depression had a 3-year delay of progression to AD if they had been treated with selective serotonin reuptake inhibitors for more than four years [76]. Therefore, treatment of NPS in preclinical AD may potentially offer an alternative target for future therapeutics, which requires further research.

We found several key methodological differences and limitations that have led to significant heterogeneity between studies. First, there was a lack of consistent definition for “cognitively unimpaired” subjects among the studies. While the majority of studies have used neuropsychological batteries with established norms, five studies (26.3%) have used predefined normal MMSE or CDR scores as surrogates. Notably, the MMSE lacks sensitivity to detect subtle cognitive symptoms or MCI [77]. As such, subjects selected on this criterion may not be representative of the cognitively unimpaired, preclinical AD populations. The inclusion of symptomatic subjects who may be at a more advanced pathological state could consequently have led to an overestimation of the associations with AT(N) biomarkers.

Second, we found significant variability in the measurement and analysis of NPS among the studies. In the 14 studies examining depression or depressive symptoms, few analyzed the clinical diagnosis of depression as a dichotomous variable, while others investigated the depressive symptoms as a continuous variable, which has excluded subjects with clinical depression. There was also a variety of self-reported scales used to measure depressive symptoms, such as the GDS, GDS-S, HADS and CES-D, making it difficult to make direct comparisons among the studies. Further, few studies have incorporated the natural history of depressive symptoms, differentiating chronic and/or recurrent symptomatology from new onset symptomatology. In studies investigating multiple domains of NPS, the NPI-Q is the most frequently used tool to measure NPS. While the NPI is an established tool that was originally designed for individuals with dementia [41], it has not been validated for the measurement of NPS in cognitively unimpaired or preclinical AD subjects. Given the recent proposal of MBI as a neurobehavioral syndrome to identify individuals at risk of incident cognitive decline and dementia [35], future studies should standardize the evaluation of NPS in pre-dementia populations by using the MBI-C [37], which has been developed specifically as an instrument for MBI case ascertainment and validated in non-demented older adults.

A clear gap in the existing literature is the lack of longitudinal data. This is highlighted by the fact that while initial cross-sectional studies from the HABS cohort showed no correlation between Aβ and NPS, longitudinal follow-up studies have shown positive findings [46, 53, 57, 58]. In addition, the duration of follow-up in most studies was 5 years or less, which may affect their ability to detect cognitive decline to MCI or dementia, given that the subjects could be in the preclinical AD stage of the AD continuum. Only one study, in which the mean duration of follow-up was 11 years, has found an association among  incident dementia, depressive symptoms and Aβ, though the use of plasma amyloid as a biomarker was a limitation [56].

Reviews on the association between AD and NPS have provided insights into the biological underpinnings of NPS as clinical manifestations of AD [19, 69, 78]. The development of AD biomarkers and neuroimaging methods has further advanced the understanding of the mechanisms leading to NPS in AD. In these reviews, brain networks/circuits that are involved in agitation, apathy and delusions [71], neuropathology, neurotransmitter, neuroimaging, ApoE genotype, inflammation, and clusterin biomarkers associated with agitation and aggression in AD [79], and metabolic dysfunctions associated with NPS in the AD continuum [70], have been discussed. Most recently, a systematic review has further shown inconsistent associations between AT(N) biomarkers and NPS such as depression, anxiety, apathy, agitation, irritability and night-time behavioral disturbances in MCI and AD dementia [44]. Similarly, the authors have found significant heterogeneity and methodological limitations that likely have contributed to the ambiguity of the overall evidence. The different definitions of cognitively unimpaired and preclinical diseases, the variable PET ligands and CSF assays, the different assessment approaches for NPS including self- and informant-rated tools with varied time reference ranges, and the use of several different analytical approaches make it challenging to reconcile and generalize the results. However, as an extension of previous reviews, the present review has provided a comprehensive summary of the existing evidence concerning the relationship between AT(N) biomarkers and NPS in preclinical AD and cognitively unimpaired subjects. We further highlighted the knowledge gap on the associations between AT(N) biomarkers and NPS in preclinical AD, provided recommendations for harmonized methodologies and suggested avenues for future research in this field.

## Conclusion

While the current evidence supports a relationship between Aβ pathology and NPS in preclinical AD and cognitively unimpaired subjects, evidence for the association between tau pathology and neurodegeneration remains unclear. There is significant heterogeneity in methodology, AT(N) biomarker and NPS among the reviewed studies. Future longitudinal studies with larger cohorts and harmonized methodologies are required not only to determine the relationship among AT(N) biomarkers, NPS/MBI and cognitive decline, but also to elucidate the contributions of comorbid pathology to preclinical AD.

## Data Availability

Not Applicable.

## Abbreviations

AD: Alzheimer’s disease
ADNI: Alzheimer’s Disease Neuroimaging Initiative
AES: Apathy Evaluation Scale
AIBL: Australian Imaging, Biomarker & Lifestyle Study of Ageing
Aβ: Amyloid-β
CES-D: Center for Epidemiological Studies Depression Scale
CSF: Cerebrospinal fluid
DIAN: Dominantly Inherited Alzheimer Network
DVR: Distribution volume ratio
FDG: Fluorodeoxyglucose
GDS: Geriatric depression scale
GDS-S: Geriatric depression scale short form
HABS: Harvard Aging Brain Study
HADS: Hospital Anxiety and Depression Scale
MBI-C: Mild Behavioural Impairment Checklist
MCI: Mild cognitive impairment
MCSA: Mayo Clinic Study of Aging
NACC: National Alzheimer’s Coordinating Center
NPI: Neuropsychiatric Inventory
NPI-Q: Neuropsychiatric Inventory Questionnaire
NPS: Neuropsychiatric symptom
OR: Odds ratio
PET: Positron emission tomography
ptau181: Phosphorylated tau181
SCD: Subjective cognitive decline
SUVR: Standardized uptake value ratio
